# PRRDB: A comprehensive database of Pattern-Recognition Receptors and their ligands

**DOI:** 10.1186/1471-2164-9-180

**Published:** 2008-04-18

**Authors:** Sneh Lata, Gajendra P S Raghava

**Affiliations:** 1 Institute of Microbial Technology, Sector39A, Chandigarh, India

## Abstract

**Background:**

Recently in a number of studies, it has been demonstrated that the innate immune system doesn't merely acts as the first line of defense but provides critical signals for the development of specific adaptive immune response. Innate immune system employs a set of receptors called pattern recognition receptors (PRRs) that recognize evolutionarily conserved patterns from pathogens called pathogen associated molecular patterns (PAMPs). In order to assist scientific community, a database PRRDB has been developed that provides extensive information about pattern recognition receptors and their ligands.

**Results:**

The current version of database contains around 500 patterns recognizing receptors from 77 distinct organisms ranging from insects to human. This includes 177 Toll-like receptors, 124 are Scavenger receptors and 67 are Nucleotide Binding Site-Leucine repeats rich receptors. The database also provides information about 266 ligands that includes carbohydrates, proteins, nucleic acids, glycolipids, glycoproteins, lipopeptides. A number of web tools have been integrated in PRRDB in order to provide following services: i) searching on any field; ii) database browsing; and iii) BLAST search against the pattern-recognition receptors. PRRDB also provides external links to standard databases like Swiss-Prot and Pubmed.

**Conclusion:**

PRRDB is a unique database of its kind, which provides comprehensive information about innate immunity. This database will be very useful in designing effective adjuvant for subunit vaccine and in understanding role of innate immunity. The database is available from the URL's in the Availabiltiy and requirements section.

## Background

More than 20 million premature deaths occur every year in the world due to infectious diseases. Every year billions of dollars are spent for the treatment of patients suffering from such diseases, which in turn, poses a great economic burden on the developing nations. Thus, protection of mankind from these dreaded diseases is one of the major challenges in the present era. Fortunately, we have effective vaccines against a number of diseases (e.g. smallpox, polio), which not only save millions of lives but also endows long lasting immunity against these diseases. But these vaccines are available only against a few diseases and the vaccines against the other infectious diseases like malaria, tuberculosis are still wanted. So, developing effective and cheap vaccines against all the infectious diseases is the need of the hour. Strategies to develop vaccines have changed tremendously over the time i.e. from whole pathogen to antigens and from antigens to antigenic regions (epitopes). Vaccines developed using antigens and antigenic regions are called subunit vaccines. They are safer and have more advantage than the vaccines based on whole organisms. Tremendous efforts have been made in the field of subunit vaccines. However, only very limited success has been achieved so far, as epitopes/antigens alone fail to elicit a robust and sustained immune response, thereby defeating the main goal of vaccination i.e. to provide strong and long lasting immunity. Therefore, in order to elicit an optimal immune response against a pathogen, there is a need to stimulate innate and adaptive immune response simultaneously [1-4]. Innate immune system has ability to recognize the pathogens using evolutionarily conserved patterns of pathogens called pathogen associated molecular patterns or PAMPs [5,6]. This natural potential of innate immune system has successfully been exploited and it is observed that addition of such microbial components to experimental vaccines leads to development of robust and durable adaptive immune response. It has now been realized that purified and synthetic components of microbial extracts or PAMPs exert potent adjuvant effects [7-9]. Thus both antigen/epitope and adjuvant are equally important for vaccine design.

Most of the effects of the above mentioned PAMPs now appear to be mediated by receptors belonging to a class of innate immunity receptors called pattern recognition receptors or PRRs. Pattern-recognition receptors (PRRs) including non-phagocytic receptors, such as Toll-like receptors, Nucleotide Oligomerization Domain (NOD) proteins and receptors that induce phagocytosis, such as Scavenger receptors, mannose receptors and β-glucan receptors. PRRs when stimulated by PAMPs lead to activation of adaptive antigen-recognition receptors subsequently inducing the expression of key co-stimulatory molecules and cytokines [2] as well as maturation and migration of other cells. Expression of co-stimulatory molecule on the surface of antigen presenting cells is absolutely necessary to flag the antigen presented by the same antigen presenting cells, as being of microbial origin and activation of antigen specific T cells. In tandem, this creates an inflammatory environment that leads to the establishment of the adaptive immune response [3,4]. So, understanding the molecular mechanism responsible for the recognition of PAMPs and generation of downstream signaling would be crucial for the development of new approaches to vaccine formulation and immunotherapy.

In this study, an attempt has been made to collect and compile the information from literature related to innate immunity for the first time. We mainly collected pattern recognition receptors (PRRs) of innate immunity and evolutionary conserved patterns of pathogens recognized by innate immunity called pathogen associated molecular patterns (PAMPs). Though the information about the PRRs may be fetched from other source like Swiss-Prot etc., the information about the more important PAMPs are scattered in the literature. So we have exerted to collect the information about PAMPs or other PRR ligands and tried to make them available from a single source. The detailed information about the PAMPs include the name of the PAMP (PRR ligand), receptor it binds to, its source, its origin and the most important, the chemical structure of the ligand (a textual description is provided in case the structure is not available). The ligands collected include the natural ligands as well as their modified derivatives like LPS (natural lipopolysaccharide) and MPL (modified derivative of LPS). This information in form of a database will be very useful for understanding the innate immune system and developing tools for predicting effective adjuvants. To the best of author's knowledge, no such database exists till date.

## Database Description

**Figure 1:**
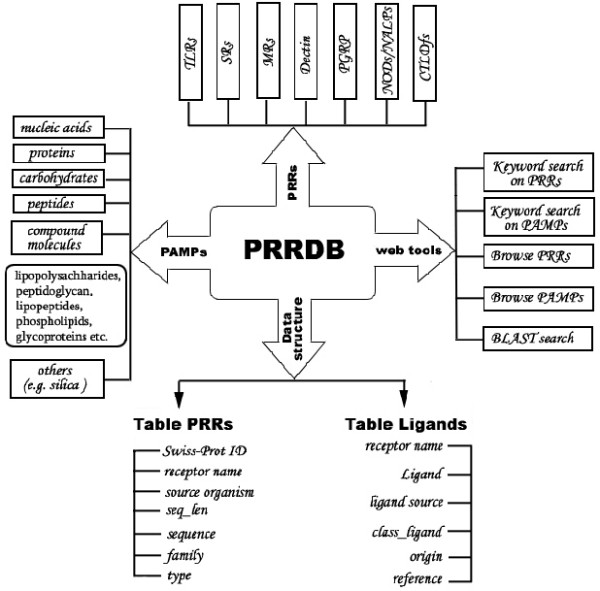
**A schematic representation of database PRRDB**. This figure gives a detailed architecture of database PRRDB. On the bottom side of the figure, tables of the database and fields of respective table are listed. On the top of the figure are the names of pattern-recognition receptors for which information is present in PRRDB. The left side provides a listing of the various types of PAMPs included in the database and the right side gives various web tools available for searching and browsing the database.

PostgresSQL relational database management system (RDBMS) has been used for storing, retrieving and managing the data. All the scripts have been written using programming language PERL. CGIperl is used for common gateway interface and Pgperl for accessing information from PostgreSQL. The server PRRDB has been developed and launched on SUN machine T1000 under Solaris 10.0 environment using Apache server. The aim of PRRDB database is to provide; i) comprehensive information about the PRRs and their ligands, ii) tools for extraction and analysis of this information and, iii) hyperlinks to related databases. The overall architecture of PRRDB is shown in Figure 1. The database consists of two tables name PRRs and ligands. The detailed description of both the tables is as follows:

### PRRs Table

This table provides comprehensive information about the pattern recognition receptors. It contains around 500 PRR sequences belonging to 77 distinct organisms, ranging from insects to humans. Out of these 177 are Toll-like receptors, 124 are Scavenger receptors, 20 are mannose receptors, 41 are C-type lectin like domain containing receptors, 17 are DC-SIGN receptors, 43 are Peptidoglycan recognition receptors and 67 are Nucleotide binding site-Leucine rich repeats rich receptors. Information about the PRRs includes their common names, synonymous names, organisms they belong to, sequence length and protein sequence. These receptors are further classified as single-pass type I/II membrane proteins, multi-pass membrane proteins, cytoplasmic or secreted proteins depending upon their site of expression. Each entry also bears a Swiss-Prot ID that is hyperlinked to its corresponding entry in Swiss-Prot. In this database we kept only well annotated PRR's with complete sequence not protein fragment or protein from cDNA libraries.

### Ligand Table

This table provides information about 266 ligands of PRRs. It provides complete information about ligands that include ligands name, source, binding receptor and its origin i.e. exogenous/endogenous. A total of 86 ligands out of 266 are endogenous in origin whereas the rest belong to the exogenous sources. The exogenous ligands may be bacterial, mycobacterial, fungal, viral, environmental or synthetic in nature. PRRDB cover a wide range of ligands, which are further classified into carbohydrates, proteins, nucleic acids, glycolipids, glycoproteins, lipopeptides, lipopolysaccharides, lipoteichoic acids, peptidoglycans, phospholipids, synthetic and others (ligands which could not be assigned to any group). A reference field is also added to the database where links are maintained to the relevant literature (Pubmed).

## Web tools

PRRDB also provides a number of online tools that allow users to retrieve and analyze the information. These web tools have been designed to facilitate the user in retrieving information from the database.

### Keyword search for PRRs

**Figure 2:**
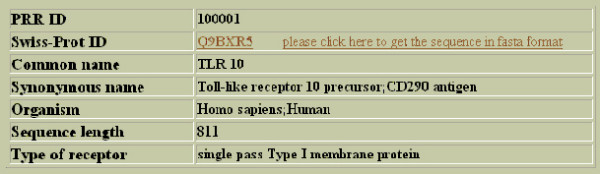
**Output of keyword search on receptors**. Typical display of output result for keyword search performed on receptors. Here a search for TLR 10 was performed and all fields were selected for display.

Keyword search option allow users to perform a search on all fields of the database. A user can also perform a specific and precise query just by entering a keyword and selecting the field in which the keyword shall be looked for. The result generated in response to a query carries along with the detailed description, the Swiss-Prot ID of that entry which is hyperlinked to its corresponding Swiss-Prot information page (Figure 2). If a user wishes to get the sequence of the receptor sequence in fasta format, he can click the hyperlink provided.

### Keyword search for ligands

**Figure 3:**
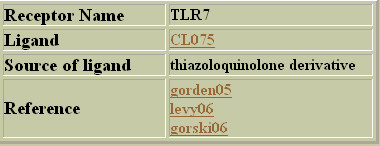
**Output of PRRDB database for keyword search on ligands**. An example of output result for keyword search on ligands performed in PRRDB. A search was performed for getting the information about the ligand CL075.

**Figure 4:**
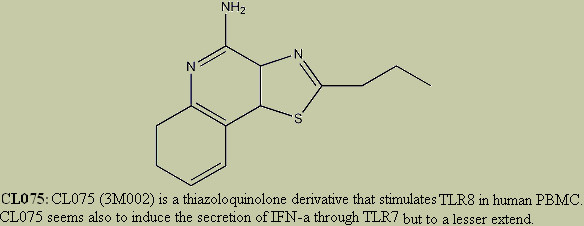
**The chemical structure of ligand CL075 as displayed in PRRDB**. Ligands in PRRDB are linked to their chemical structures (if available) or their brief textual description. Therefore, in this case, clicking on ligand CL075 would direct you the chemical structure of this ligand.

This option works just as stated above, but for the ligands (Figure 3). The names of the ligands are hyperlinked to an html page displaying the chemical structure of the ligand (Figure 4) if it is available otherwise a brief description about ligand is provided. The reference field is also present where links are maintained to the relevant literature (mainly Pubmed) whenever that is readily present.

### Browse PRRs

PRRDB provides browsing PRRs facility where users may browse a group or set of receptors. For example user may display all Toll-like receptors stored in PDRB by a single click. One of the major advantages of browsing over keyword search is that users may examine receptors even if they do not know appropriate keyword.

### Browse Ligands

Similar to "Browse PRRs", PRRDB also provides facility for browsing ligands where user may browse different type of ligands. Browsing of ligands includes browsing of following type of ligands carbohydrates, proteins, Nucleic acids, peptides, lipoproteins etc.

### BLAST search

One of the powerful features of PRRDB is similarity search. Users may search their protein sequence against known PRRs in PRRDB for similarity search using BLAST [10]. This allows user to annotate their query protein. If the query protein has high similarity with any receptor sequence in PRRDB than user may annotate his protein. This is very useful features for checking whether a given sequence is a PRR.

## Potential utility and limitations

In the past few years research in the field of subunit vaccine designing has been focused mainly on searching for potential candidates or epitopes that could be used as antigens. For this reason a large number of bioinformatics databases [11-17] and tools [18-23] have been developed for predicting antigenic epitopes or antigens. Recent studies clearly indicate that it is not the antigens alone but a combination of antigen and adjuvant that is required to optimally elicit an immune response. There is a need to activate adaptive as well as innate immune system. This database would help the bioinformaticians and immunologists to understand the interaction between PRRs and PAMPs. Apart from the comprehensive information about the PRRs that may be available elsewhere this database also houses information about the PRR ligands collected from the published literature. Collection of PAMPs at one single place has never been done before. The database provides, along with other important details, the chemical structures of the ligands (or textual description in case structure is not available). As these PAMPs belong to a wide range of organisms, the representative structure is given e.g. if TLR4 binds to lipid A that may be present in any of the gram-negative bacteria, then the structure of E. coli lipid A is given. This would help the scientists to look into the fine structural details that are required for a PAMP to act as an adjuvant. One of the main problems for using PAMPs as adjuvants is the toxic effect exhibited by most of them. Therefore, the ligands collected include the information about a parent PAMP that shows toxic effect when used as adjuvant as well as its modified derivatives that is much safer to use. For example, it is known that LPS binds to TLR4 and can act as an adjuvant but it is toxic and pyrogenic. Whereas, its modified derivative MPL is non-pyrogenic and retains the TLR4 activating property. Examples like these would help the scientists to look into the structural details and study what is the minimal region required for the desired activity and is safer at the same time. Briefly, the information available at PRRDB will be very useful for immunologist to understand immune system and in designing novel and improved subunit vaccines. Major limitation of this database is that it has limited information, not suitable for deriving the generalized rules. We hope, over the time information on innate immunity will increase and so would grow the size of the database. This is the first attempt towards understanding pathogen/ligands recognition mechanism of innate immune system and for rational ligands/adjuvants designing.

## Availability and requirements

PRRDB is available at: 

or: 

## Authors' contributions

SL collected and annotated the data from literature and existing databases. SL also created tables in PostgreSQL and developed web server. GPSR conceived the project, coordinated it and refined the manuscript drafted by SL.
